# Lateral Condyle Fracture of the Humerus in Children Treated with Bioabsorbable Materials

**DOI:** 10.1155/2013/869418

**Published:** 2013-10-09

**Authors:** Véronique Andrey, Stéphane Tercier, Frédéric Vauclair, Aline Bregou-Bourgeois, Nicolas Lutz, Pierre-Yves Zambelli

**Affiliations:** Unité Pédiatrique de Chirurgie Orthopédique et Traumatologique (UPCOT), Lausanne University Hospital (CHUV), Site de l'hôpital de l'enfance, Avenue Montétan 16, 1007 Lausanne, Switzerland

## Abstract

The aim of this study was to compare clinical and radiological outcome of lateral condyle fracture of the elbow in children treated with bioabsorbable or metallic material. From January 2008 to December 2009, 16 children with similar fractures and ages were grouped according to the fixation material used. Children were seen at 3, 6, and 12 months and more than 4 years (mean 51.8 months) postoperatively. The clinical results were compared using the *Mayo Elbow Performance Score* (MEPS). Radiographic studies of the fractured and opposite elbow were assessed at last follow-up control. Twelve children had a sufficient followup and could be included in the study. Seven could be included in the traditional group and 5 in the bioabsorbable group. At 12 months, the MEPS was 100 for every child in both groups. Asymptomatic bony radiolucent visible tracks and heterotopic ossifications were noted in both groups. There were no significant differences in terms of clinical and radiological outcome between the two groups. The use of bioabsorbable pins or screws is a reasonable alternative to the traditional use of metallic materials for the treatment of lateral condyle fracture of the elbow in children.

## 1. Introduction

After supracondylar fracture, distal humerus epiphyseal fracture is the second most frequent injury of the elbow in children. Epiphyseal fractures of the distal humerus are described in relation to their location. The lateral condyle is by far the more frequent. The severity of the fracture is graded from 1 to 3. A fracture without displacement is graded 1 and treated conservatively. Grades 2 and 3 represent moderate and severe displacement, respectively, and need a surgical approach [1–3]. Traditional surgical treatment consists of an open anatomical reduction, metallic Kirchner wire fixation, and cast immobilization. The metallic hardware is usually removed 6 to 8 weeks later under general anesthesia [4, 5].

In the 90s, the first bioabsorbable materials made of polyglycolic acids were used in traumatic and orthopedic surgery. Because of strong inflammatory reaction and significant clinical side effects (osteolysis, seroma formation), the use of traditional materials remained the gold standard [6, 7]. New bioabsorbable materials made of polylactic acids were introduced. They resorb slower and do not induce clinically disturbing inflammatory reactions [8]. Many orthopaedic and trauma studies confirmed the safety and efficacy of these newer bioabsorbable materials without significant side effects in adults [8–11] and with similar clinical outcome, when compared to traditional metallic materials [12–14]. In 1991, a study assessing polyglycolic bioabsorbable materials for the treatment of epiphyseal fractures of the distal humerus did not reveal significant side effects or growth disturbances after 6 months although aspecific inflammatory reactions were noticed [15–17]. The use of polylactic bioabsorbable materials did not show any bony abnormalities after one to two years, but suggested that a minimal 3 years followup was necessary to ascertain the absence of any impact on the growing bone [18, 19]. In our hospital since 2009, metallic K-wires were replaced by bioabsorbable polylactic acid materials. Since polylactic materials have a significantly longer resorption time than polyglycolic materials, their impact on growing bone needed to be further assessed. 

The aims of this study were to demonstrate that the use of polylactic bioabsorbable materials in lateral condyle fractures of distal humerus in children did not significantly impair the growing elbow and that the functional outcome was as good as with traditional metallic materials. 

## 2. Materials and Methods

From January 2008 to December 2009, 16 children underwent surgical treatment of a lateral condyle fracture of the elbow in our pediatric orthopaedic and trauma unit. The first group (group 1) consisted of 10 children operated in 2008 using traditional metallic K-wires for fixation after open anatomical reduction. Each child required a second operation for hardware removal 6 to 8 weeks after trauma.

In 2009, 6 children with similar fractures constituted group 2 and were treated using bioabsorbable pins and/or screws with the same surgical approach. 

Each patient was operated by the same team of senior surgeons using the following surgical technique.

### 2.1. Surgical Technique

The operation was performed under general anesthesia on the day of injury or the day after.

In group 1, once open anatomical reduction was achieved and confirmed using fluoroscopy, fixation was secured using one or two 1.0 to 2.0 millimeter transepiphyseal metallic K-wires. Skin closure covered the wires. Postoperatively, the elbow was immobilized in a long arm cast for 1 month. The hardware was removed under general anesthesia after 6 to 8 weeks.

In group 2, open anatomical reduction was temporary stabilized with metallic K-wires until final fixation with polyglycolic bioabsorbable wires and/or screws. Skin was closed after hardware removal. The bioabsorbable wires were 2.0 millimeters in diameter and had an estimated resorption time of 24 months. The elbow was also immobilized in a long arm cast for 1 month.

A retrospective analysis of both functional and clinical outcomes was performed during the regular followup after 3, 6, and 12 months and more than 4 years after surgery. The functional outcome was evaluated according to the calculated* Mayo Elbow Performance Score *(MEPS) [20, 21]. Medical records were searched for possible clinical, operative, and postoperative complication. For the purpose of the study, AP and lateral plain radiographic studies of the fractured and contralateral healthy elbows were performed at one and four years after fracture fixation. Radiographic assessment looked for bony abnormalities such as radiolucent visible tracks, heterotopic ossifications, or bony cysts. Growth plate disturbances were recorded. When disagreement was noted among the authors' interpretation, the films were reviewed in common and agreement was reached. Baumann's angle was measured and compared with the healthy side to evaluate the quality of the reduction. Valgus or varus deformity was considered significant if more than 10 degrees. Elbow range of motion (ROM) was considered significantly impaired when 20 or more degrees loss was noted in flexionextension.

Radiological abnormalities and clinical complications were listed and analyzed in both groups. The continuous variables, clinical scores, and Baumann's angle differences were evaluated between the two groups using the Wilcoxon's test for unpaired samples. 

## 3. Results

Three children in group 1 and one in group 2 moved away and were lost to followup. The remaining 7 children in group 1 were 2 girls and 5 boys with a mean age of 9,2 years (range: 5–14). The 5 children in group 2 were 2 girls and 3 boys with a mean age of 7,7 years (range: 5–14). Demographic data are listed in Table 1.

After four years, no seroma, discharging sinus over the fracture site or osteolytic changes was noted in the bioabsorbable group. In both groups, no infection, loss of fracture reduction, avascular necrosis, or pseudarthrosis occurred. 

At the final follow-up control, significant valgus deformity of more than 10° was noted in 1 case for group 1 and 2 cases for group 2. These 3 cases remained clinically asymptomatic. 

Less than 20° decrease in the elbow ROM, without any expressed functional consequences, was measured in four cases in group 1 and three cases in group 2 (Table 2). One patient in group 2 had a 35° loss of ROM on the fractured side without expressed functional consequences at one-year followup. Complementary investigations with a CT-scan revealed heterotopic calcifications over the coronoïd process. He benefited from a second procedure with heterotopic calcification removal. One-year after the second operation, his fractured elbow flexion limitation reduced to 10°. 

Regarding functional outcome, the mean MEPS at 1 month was 75 for each patient in both groups and was considered to be secondary to the long cast immobilization. At 3 months, the mean MEPS was 95,7 in group 1 (range 85–100) and 95 in group 2 (range 90–100). At 6 months, the mean MEPS was 99.2 in group 1 (range 95–100) and 99 in group 2 (range 95–100). The score reached 100 in each patient of both groups at one-year followup and after. There was no statistically significant difference between the 2 groups' mean scores at 3, 6, and 12 months (Table 3).

The MEPS's reduction in both groups was mostly due to mild or moderate pain and decreased ROM. Of note, each child from both groups was free of pain at one-year followup and had returned to his normal activities.

**Figure 1:**
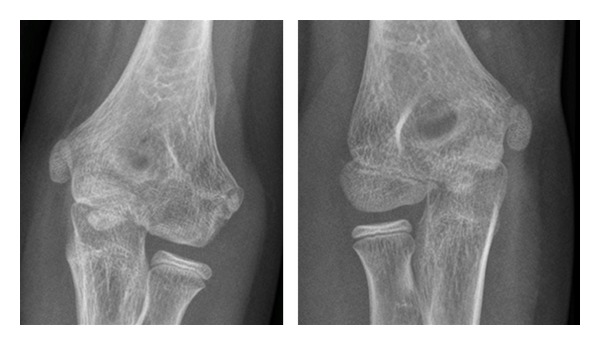
Fractured elbow compared to the contralateral healthy elbow at 4-year followup (group 1).

**Figure 2:**
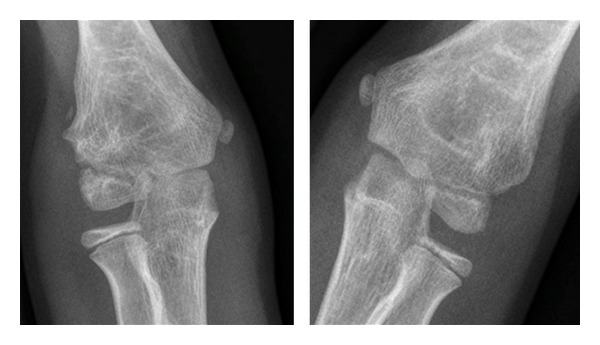
Fractured elbow compared to the contralateral healthy elbow at 4-year followup (group 2).

When comparing normal and operated elbow radiographs at four years, two cases of condylar bone remodeling were observed in group 1 and one case in group 2 (Figures 1 and 2). Two cases of heterotopic ossifications without significant functional consequences were observed in both groups. Two patients in group 2 had clinically nonsignificant persistent visible radiolucent bony tracks at one-year followup. As previously explained only one patient in group 2 needed complementary investigations with a CT-scan because of heterotopic calcifications. In group 1, one case of premature growth plate closure occurred (Table 2).

At one-year followup, no epiphyseal necrosis was noticed on radiographs.

Baumann's angle difference between the healthy and operated elbows was a mean 2.7° (range 0–6) in group 1 and 8.6° (range 0–18) in group 2. This difference did not reach statistical significance (Table 3).

## 4. Discussion

The gold standard in the treatment of displaced lateral condyle fractures of the elbow in children is open anatomical reduction and internal fixation with K-wires followed by cast immobilization [4, 5]. Although very effective with excellent functional results and few complications, this technique implies for some surgeons the need for hardware removal under GA. Injured children with this condition would greatly benefit from any bioabsorbable material giving similar results. 

In this study, the functional outcome was excellent and identical in both groups more than 4 years after surgery. Twelve months after fixation, the MEPS reached 100 in every patient of both groups. Compared to a previous study performed with polyglycolic materials revealing cases of nonspecific inflammatory reactions like seroma formation [17], no such significant inflammatory process was noticed in our patients. 

Using bioabsorbable pins and screws requires fine technical skills and good knowledge of the material, especially when applied to a small size elbow. Once anatomically reduced, the fracture needs to be stabilized by metallic K-wires until final fixation with the bioabsorbable material. Because of these manipulations and the small intraoperative surgical space available, secondary displacement may occur. It could explain the slight increased of deformation or limitation of ROM observed in the bioabsorbable group. Of note, these findings were not clinically significant and did not influence the function of the elbow at one-year followup. In this small series, no clinical complications could be directly attributed to the use of bioabsorbable materials. In the literature, ROM limitations and valgus or varus deformities are usual complications reported after lateral condyle fracture of children's elbow, irrespective of the fixation technique [22, 23].

Radiographic bony abnormalities such as heterotopic ossifications along the fracture site or bone remodeling were noted one year after surgery for both techniques. These findings were clinically irrelevant except for one patient from group 2. This 14-year-old child had a limited elbow ROM of 35° degrees 12 months after surgery. After computed tomography evaluation he was reoperated 12 months after fracture fixation. However, this patient was never symptomatic before the second procedure. One year later, the ROM improved significantly to less than 10° flexion loss and his functional outcome was excellent.

One case of premature closure of the growth plate was noted in an 11-year-old child in group 1, without clinical and functional consequences. No difficulty was encountered during the surgical procedure. The advanced bone age compared to his chronological age enabled healing without significant malunion, with the contralateral healthy growth plate being almost closed at the time of injury. 

Although Baumann's angle measurements were variable among examiners, especially for older cases where the capitellum starts to fuse with the lateral condyle, there was no significant difference between both groups.

This study had naturally some limitations. It was a retrospective analysis with a small sample size. Radiographic analysis was performed independently by the authors and only Baumann's angle was measured on radiographics. 

In our study, bioabsorbable screws and pins did not induce any significant radiological growth disturbances or abnormal bone reaction. In accordance with previous studies using similar material in orthopedic surgery, children operated with bioabsorbable materials need a minimum of three-year followup to confirm the absence of complications such as foreign body reaction and cysts formation [18, 19].

As functional results were similar using both techniques, the benefits of using bioabsorbable material were clear. A second operation is avoided which widely compensates for the initial higher cost of the bioabsorbable material. 

## 5. Conclusion

When compared to metal fixation, bioabsorbable fixation of lateral condyle fractures of the elbow was safe. It also is costeffective when for hardware removal, a second anaesthetic is planned.

No clinically relevant specific complication or adverse reaction could be attributed directly to the bioabsorbable material. More than four years after surgery, the functional outcome was excellent. Nonsignificant radiographic bone modifications around the fracture were noted in both groups. Using bioabsorbable material for the surgical treatment of lateral condyle fractures of the elbow appeared as a satisfying alternative to metal K-wires. 

## Figures and Tables

**Table 1:** Patient characteristics: Age, gender, and side of injury.

Case	Gender	Age at time of injury	Side of injury
(Group 1)			
1	F	6	G
2	M	6	G
3	F	5	G
4	M	14	G
5	M	7	G
6	M	5	D
7	M	11	G

(Group 2)			
1	F	14	G
2	M	14	G
3	M	6	G
4	M	5	D
5	F	7	G

**Table 2:** Summary of results: MEP scores and complications.

Case	Age	Mayo Elbow performance score				Complications
		1 month	3 months	6 months	After 12 months	
(Group 1)						
1	6	75	95	95	100	None
2	6	75	95	100	100	None
3	5	75	100	100	100	None
4	14	75	100	100	100	None
5	7	75	100	100	100	Valgus > 10°
6	5	75	95	100	100	None
7	11	75	85	95	100	None

(Group 2)						
1	14	75	100	100	100	None
2	14	75	90	95	100	ROM reduction
3	6	75	95	100	100	Valgus > 10°
4	5	75	95	100	100	None
5	7	75	95	100	100	Valgus > 10°

**Table 3:** Summary of results: mean age, MEP scores, and Baumann's angle variation.

Parameter	Group 1	Group 2	*P* value
Age	9,2 years (5–14)	7,7 years (5–14)	0.5011
Mayo score (1 month)	75	75	1
Mayo score (3 months)	95,7 (85–100)	95,0 (90–100)	0,427
Mayo score (6 months)	99,2 (95–100)	99,0 (95–100)	1
Mayo score (after 12 months)	100	100	1
Baumann angle variation	2,7° (0–6)	8,6° (0–18)	0.1915
